# Morphologic analysis of the proximal tibia after open wedge high tibial osteotomy for proper plate fitting

**DOI:** 10.1186/s12891-016-1277-3

**Published:** 2016-10-10

**Authors:** Oui Sik Yoo, Yong Seuk Lee, Myung Chul Lee, Jae Hong Park, Jae Won Kim, Doo Hoon Sun

**Affiliations:** 1 Central R&D Center, Corentec Co Ltd, Cheonan, South Korea; 2 Department of Biomedical Engineering, Yonsei University, Seoul, Korea; 3 Department of Orthopaedic Surgery, Seoul National University College of Medicine, Bundang Hospital, 166 Gumi-ro, Bundang-gu, Seongnam-si, Gyeonggi-do 463-707 South Korea; 4 Department of Orthopaedic Surgery, Seoul National University College of Medicine, Seoul National University Hospital, Bundang, South Korea

**Keywords:** Knee, High tibial osteotomy, Stability, Plate, Fitting

## Abstract

**Background:**

After open wedge high tibial osteotomy (OWHTO), the proximal fragment resembles the anatomy of the proximal tibia that is aligned in the anterior-posterior direction and the distal fragment resembles the anatomy of the mid shaft that is aligned in the proximal-distal direction. In addition, the medial portion of the proximal fragment becomes aligned medially and the medial portion of the distal fragment, laterally, depending on the magnitude of the posterior opening gap. Therefore, there would be a mismatch between the post-correction bony surface and the previous pre-contoured plate geometry. The purpose of this study was to devise a new plate that best fit the post-contoured anatomy of the tibia by evaluating the surface geometry of the plate positioning site after OWHTO.

**Methods:**

Thirty-one uni-planar and 38 bi-planar osteotomies were evaluated. Surgical indications were age of under 70 years, relatively active patient who performs recreational sports activities. Other indications were similar with general recommendation of HTO. Computed tomography (CT) of the operated knees was performed and it was used for the reconstruction of the 3D model. Bone model axis re-alignment was performed with coronal, sagittal, and axial plane. Morphologic analysis of the proximal tibia was performed using the following parameters: (1) radii in axial plane, 2) radii in coronal plane, and 3) angle and horizontal distance (Distance X) between the proximal and distal fragments. These were also analyzed according to the correction degree. The Analysis of Variance (ANOVA) test was conducted to verify the change depending on the correction amount of the posterior opening gap. The values obtained for the uni- and bi-planar osteotomy were compared by the independent *t*-test.

**Results:**

There were 9 male and 60 female patients were recruited to this study; the mean age was 58.3 ± 8 and 56.9 ± 7.6 years, respectively. Preoperative weight bearing line (WBL) was 21.59 ± 11.36 and 22.32 ± 10.55 %, respectively. Mean correction degree was 10.9 ± 2.7 and 11.1 ± 2.6 mm, respectively. The radii of the tibial cross-sectional contour at the head portion tended to increase from the proximal to distal direction. The radii of the tibial cross-sectional contour at the neck portion tended to decrease from the proximal to distal direction. The radii of the coronal plane tended to increase from the proximal to distal direction. The angle between the proximal fragment and the distal one varied with the correction amount of the posterior opening gap. Shaft_Mid and Distance X of GroupI (110.08 mm and 6.11 mm, respectively) which had lower correction angle were lower than those of GroupII (130.05 mm and 6.41 mm, respectively) and those of GroupIII (136.35 mm, 8.01 mm, respectively) in coronal plane. There were significant differences (*p* = 0.023 < 0.05 and *p* = 0.009 < 0.01, respectively).

**Conclusion:**

Current plate design should be modified to the surface geometry of the post-correction for the proper fitting. As the correction degree increases, the plate should be bended at the both end of the opening gap in coronal plane.

**Trial registration:**

‘retrospectively registered (ISRCTN97792440).

## Background

Open wedge high tibial osteotomy (OWHTO) is a well-established treatment for medial uni-compartmental arthritis of the knee joint [1–5]. To ensure proper load re-distribution, the osteotomy site should be opened at the medial side and the magnitude of the opening should be based on the preoperative deformity. After the correction, proper load re-distribution should be maintained during the healing process for improving the radiological and clinical outcomes. Therefore, stable fixation is needed for the safe healing of this additive type of osteotomy in order to minimize the risk of non-union and loss of correction [6].

A position HTO plate (such as a Puddu plate) is a short plate comprising an integrated spacer block, available in different sizes, corresponding to the amount of the medial opening desired [6]. However, a high plate-related complication rate and significant loss of correction have been being reported [7–10]. Recently, a long and rigid titanium plate (Tomofix, Depuy Synthes, PA, USA), which is anatomically pre-contoured to the medial tibial metaphysis, was designed specifically for OWHTO. This implant is equipped with locking bolts and, thus, functions as an internal plate fixator [6, 11, 12]. Nowadays, new anatomical locking metal block plates are available; it is also found that locking plates with an additional metal block are more stable than those without a metal block [2].

**Fig. 1:**
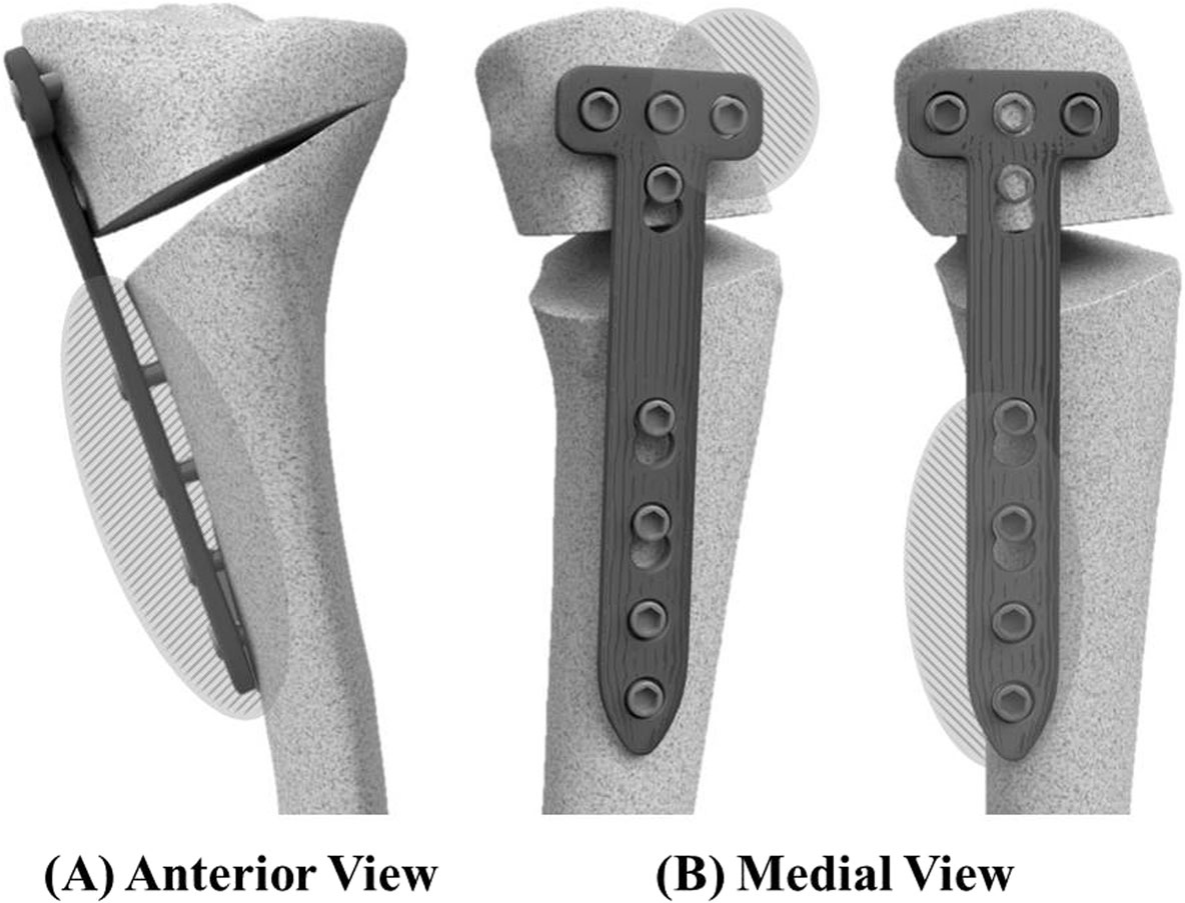
Mismatch of the OWHTO plate on the osteotomized bony surface: Mismatch of the coronal plane occurs by moving distal fragment laterally **a** and mismatch of the sagittal plane occurs by different centers between the proximal and distal fragment **b**

However, these plates are designed as a pre-contoured anatomy of the medial aspect of the proximal tibia. The proximal tibia has a unique 3-dimensional anatomy compared to the mid or distal tibia, because multiple structures including the tibia plateau, central fovea, and posterior cortex undergo changes abruptly [13–15]. After OWHTO, the proximal fragment resembles the anatomy of the proximal tibia that is aligned in the anterior-posterior direction and the distal fragment resembles the anatomy of the mid shaft that is aligned in the proximal-distal direction. In addition, the medial portion of the proximal fragment becomes aligned medially and the medial portion of the distal fragment, laterally, depending on the magnitude of the posterior opening gap (Fig. 1). Therefore, there would be a mismatch between the post-correction bony surface and the previous pre-contoured plate geometry.

The purpose of this study was to devise a new plate that best fit the post-contoured anatomy of the tibia by evaluating the surface geometry of the plate positioning site after OWHTO. The hypotheses tested in this study were as follows: (1) the anatomical plate should have an abrupt bending 3 dimensionally around the posterior opening gap and 2) the importance of this tendency increases with the increase in the posterior opening gap.

## Methods

### Demographics

From March 2012 to June 2014, 31 uni-planar and 38 bi-planar osteotomies were retrospectively evaluated. All patients underwent OWHTO for treatment of medial uni-compartmental osteoarthritis with a varus deformity. There were 9 male and 60 female patients were recruited to this study; the mean age was 58.3 ± 8 and 56.9 ± 7.6 years, respectively. The mean body mass index (BMI) was 26.2 ± 2.7 and 27.9 ± 3.9, respectively. The mean range of motion (ROM) was 132 ± 9.5 and 133 ± 11°, respectively. Preoperative weight bearing line (WBL) was 21.59 ± 11.36 and 22.32 ± 10.55 %, respectively, and mean correction degree was 10.9 ± 2.7 and 11.1 ± 2.6 mm, respectively. Preoperative demographic data of 2 groups were not statistically different (n.s.). The surgical indications were relatively active patients aged under 70 years and a varus arthritic knee. Additionally, per the general recommendations of HTO, some other conditions such as mild patellofemoral arthritis were also considered. The exclusion criteria were 1) OWHTO performed by cosmetic problem, 2) OWHTO performed by combined ligament instability, and 3) double osteotomy.

Computed tomography (CT) of the operated knees of the uni-planar and bi-planar group was performed at 2.5 days (range, 2–5 days), using CT scanners (SOMATOM Definition, Siemens, Forscheim, Germany; MX8000, Brilliance 64, Brilliance iCT, Philips, Netherland). The CT was conducted under the following conditions: tube voltage (120 kV), tube current (250 mAs), pitch (0.609), slice thickness (3 mm), and resolution (standard). A continuous scan was obtained from approximately 10 cm above to 10 cm below the joint line. Institutional review board approval was obtained prior to initiation of the study, and all patients provided informed consent for participation.

#### Surgical technique

An approximately 5 cm incision is made longitudinally at the 1 cm anterior portion of the posterior crest of the tibia. This incision is more posterior than the usual incision because it allows for easy insertion of a releaser and a protector. The interval behind the patellar tendon is now freed, and the insertion area of the tendon is protected using a retractor. Then, the superior border of the pes anserinus is incised, the medial collateral ligament is mobilized from the tibia, and release is performed by insertion of a periosteal elevator.

Release behind the posteromedial cortex of the tibia is typically done using gauge packing, which; enables access to the more than half of the posterior cortex of the tibia. After removal of the gauge, the releaser is inserted through this interval and further release is performed by pushing the releaser until making contact is made with the posterior cortex. If the tip of the releaser reaches to the fibular head area, the protector is inserted at the interval between the posterior cortex and the releaser, and the releaser is removed.

With the help of anterior-posterior C-arm image, the tip of the protector is hooked to the target portion of the hinge located at the lateral cortex of the proximal tibia. Then, the cutting block is attached to the protector and pushed to the posteromedial cortex of the tibia. If contact is made, the cutting block is tightened to the protector and guiding pins are inserted at the four holes of the cutting block [16]. Finally, sawing is performed; the main goal of this procedure is sawing of the posterior cortex. After removal of the protective cutting complex, C-arm images are checked. Finally, in the bi-planar osteotomy, anterior retrotubercle osteotomy is performed, with distraction at the most posterior gap. The amount of distraction or control of tibial slope is adjusted according to pre-operative planning and it was adjusted at the most posterior gap [17]. Usually, target point was around 62.5 % of the weight bearing line (WBL) and was adjusted from 55 to 65 % according to the status of the medial compartment. If the medial compartment showed severe degeneration, it was shifted to the larger correction of approximately 65 %.

### Evaluations

**Fig. 2:**
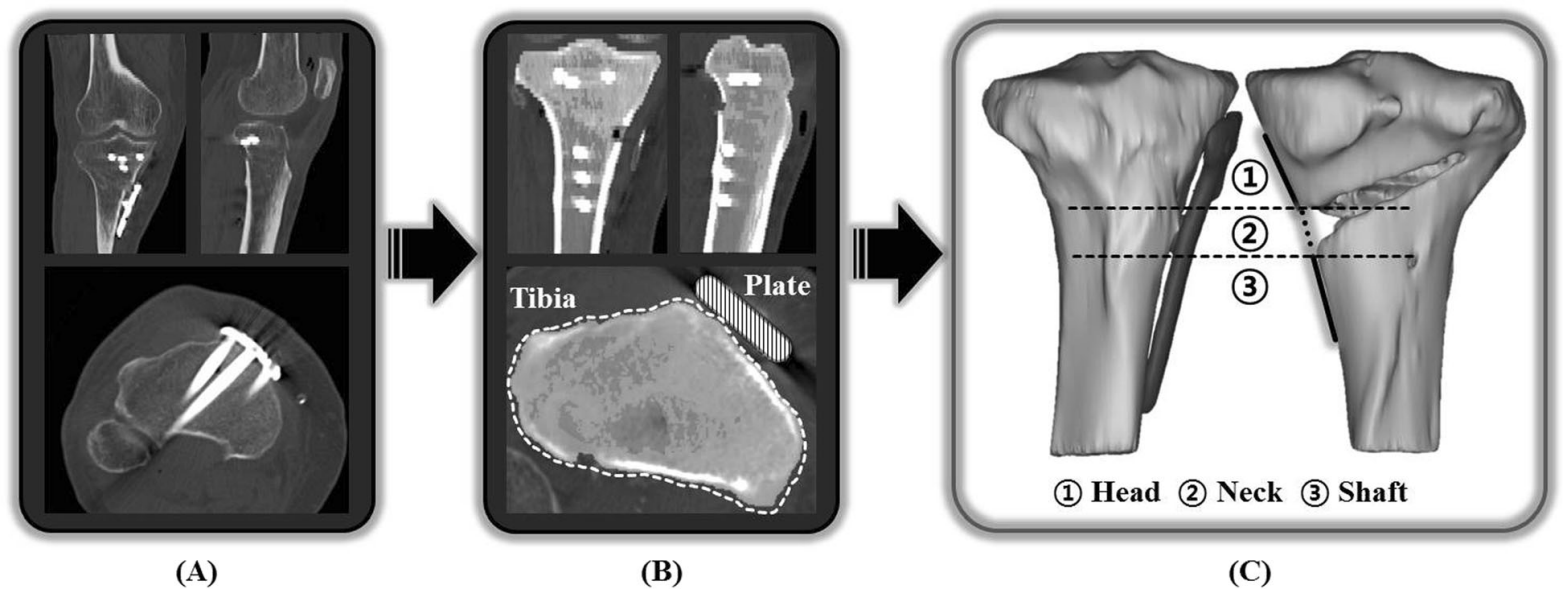
Modeling is performed by acquisition of the data **a** selection of the ROI **b** and 3D reconstruction process **c**

Postoperative CT data obtained after OWHTO using a locking plate were used for the reconstruction of the 3D model with Mimics v.16.0 (Materialise, Leuven, Belgium) of the proximal tibia and locking plate. Segmentation of the region of interest (ROI) was obtained from Digital Image Communication in Medicine (DICOM) data, and ROI was determined by adjusting and setting of Hounsfield Units (HU) threshold. Then, reconstruction was performed from the segmentation by stacking process of the contour [18, 19] (Fig. 2).

**Fig. 3:**
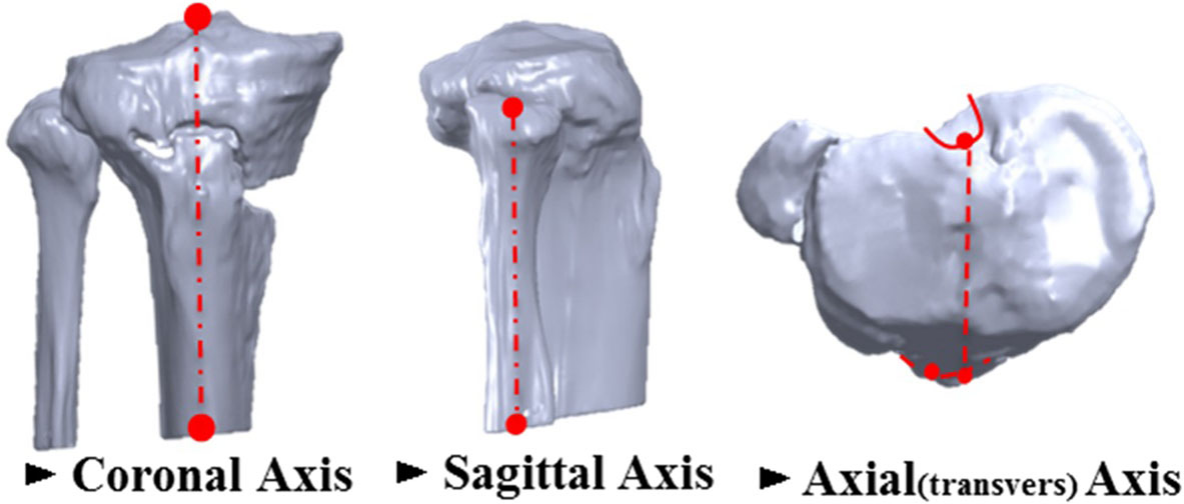
Bone model re-alignment process

**Fig. 4:**
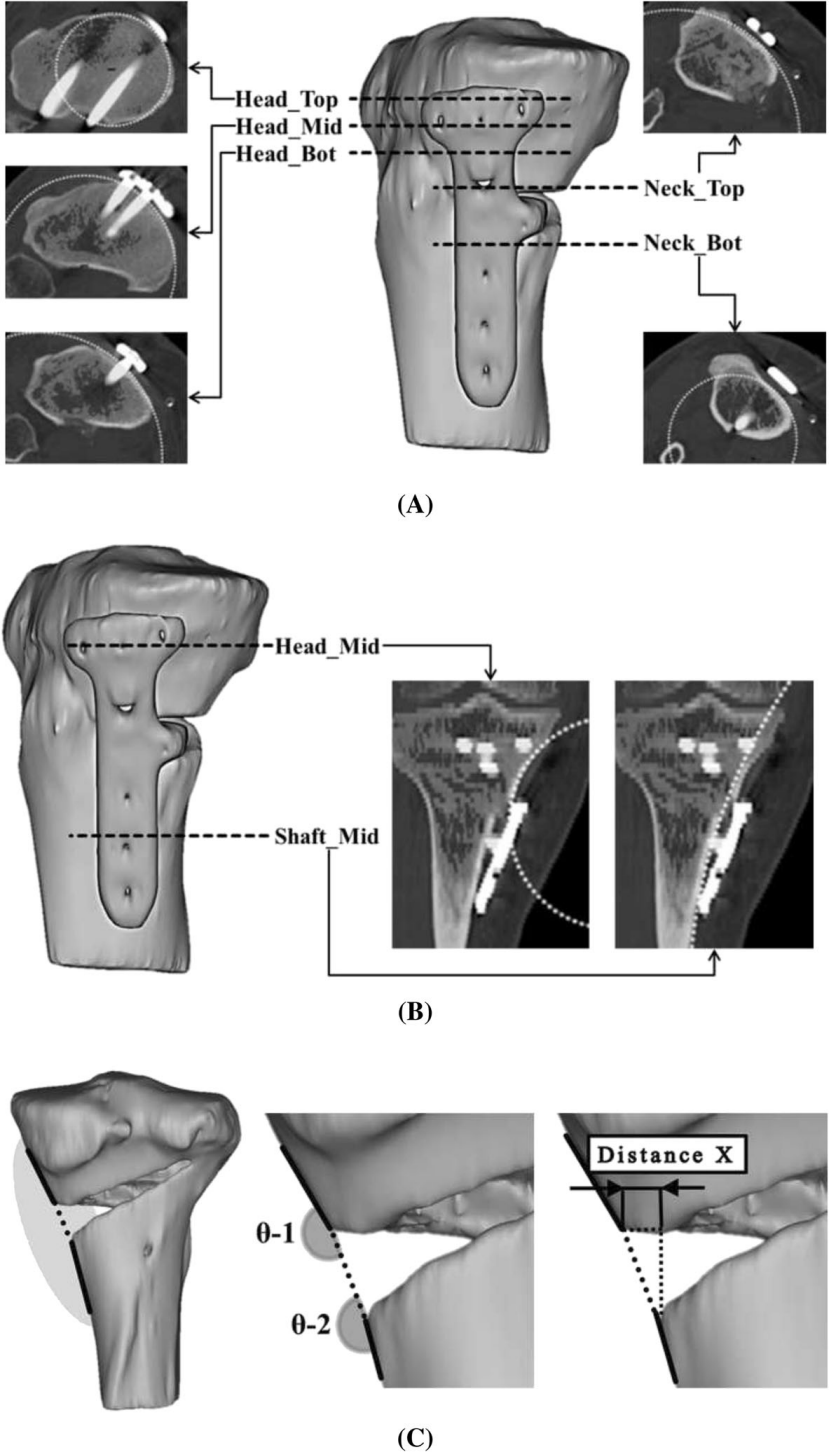
Criteria of morphologic factors in the axial plane **a** in the coronal plane **b** and the angle and horizontal distance **c**

Axis re-alignment was needed during reconstruction of the 3D model because the coordinates of the postoperative CT data were respectively different according to the posture of the patient and their progress after surgery. The coronal axis was re-aligned with the distal tibia center to the tibial spine, the sagittal axis was re-aligned in the same line with the fibula, and the axial (transverse) axis was re-aligned with the center of posterior cruciate ligament (PCL) insertion to the medial one-third of the tibial tuberosity using SolidWorks 2014 (Dassault Systemes, Waltham, Massachusetts, USA) (Fig. 3). Morphologic analysis of the proximal tibia was performed using the following parameters (Y.O.S. and K.J.W., experience of more than 5 years): (1) radii in axial plane, 2) radii in coronal plane, and 3) angle and horizontal distance (Distance X) between the proximal and distal fragments. The parameters were measured at 3 borders because the contours were underwent changes in the proximal fragment (head), gap (neck), and distal fragment (shaft). The radii in the axial plane were measured at the head and neck positions. The measurements of the radii showed that the radii increased with the increase in the flatness of the surface and the radii decreased with the increase in the surface curvature. The radii of the head in axial plane were measured at 3 positions (Head_Top: top of the implant head part, Head_Mid: middle of the implant head part, and Head_Bot: bottom of the implant head part) and those of the radii of the neck, at 2 positions (Neck_Top: top of the implant neck part and Neck_Bot: bottom of the implant neck part). The distance between each position was 3 mm (Fig. 4a). The radii of the coronal plane were measured at 2 positions. First position was measured at the middle of the head center in coronal plane (Head_Mid) and second position was measured at the middle of the shaft center in coronal plane (Shaft_Mid) (Fig. 4b). Two angles were measured at block insertion in the coronal plane: θ–1 was the angle between the contour line of the proximal fragment and the line connecting the end point of the proximal fragment and the end point of the distal fragment, and θ–2 was the angle between the contour line of the distal fragment and the line connecting the end point of the proximal fragment and the end point of the distal fragment. Horizontal distance (Distance X) was measured between the end point of the proximal fragment and the end point of the distal fragment (Fig. 4c). These data were also compared between uni-planar and bi-planar osteotomy groups. The correction for the posterior opening gap that was measured at the posterior opening gap ranged from 5 to 16 mm. Therefore, measurements were divided into 3 groups (Group1: 5–8 mm, *n* = 19, Group2: 9–12 mm, *n* = 29, Group3: 13–16 mm, *n* = 21) according to the correction amount of the posterior opening gap. Two specialist of this field measured twice with 2–3 weeks interval. The reliability of the measurements was assessed by examining the intra-rater and inter-rater reliability using the intra-class correlation coefficient (ICC).

### Statistics

Statistical analyses were performed using the SPSS statistical software v.22 (IBM, New York, USA), in which a significance threshold of 0.05 was set for all statistical comparisons. The Analysis of Variance (ANOVA) test was conducted to verify the change depending on the correction amount of the posterior opening gap. The values obtained for the uni- and bi-planar osteotomy were compared by the independent *t*-test. Sample size was calculated from following values (α error 0.05, power 0.85, effect size 0.5: sample size 31).

## Results

**Fig. 5:**
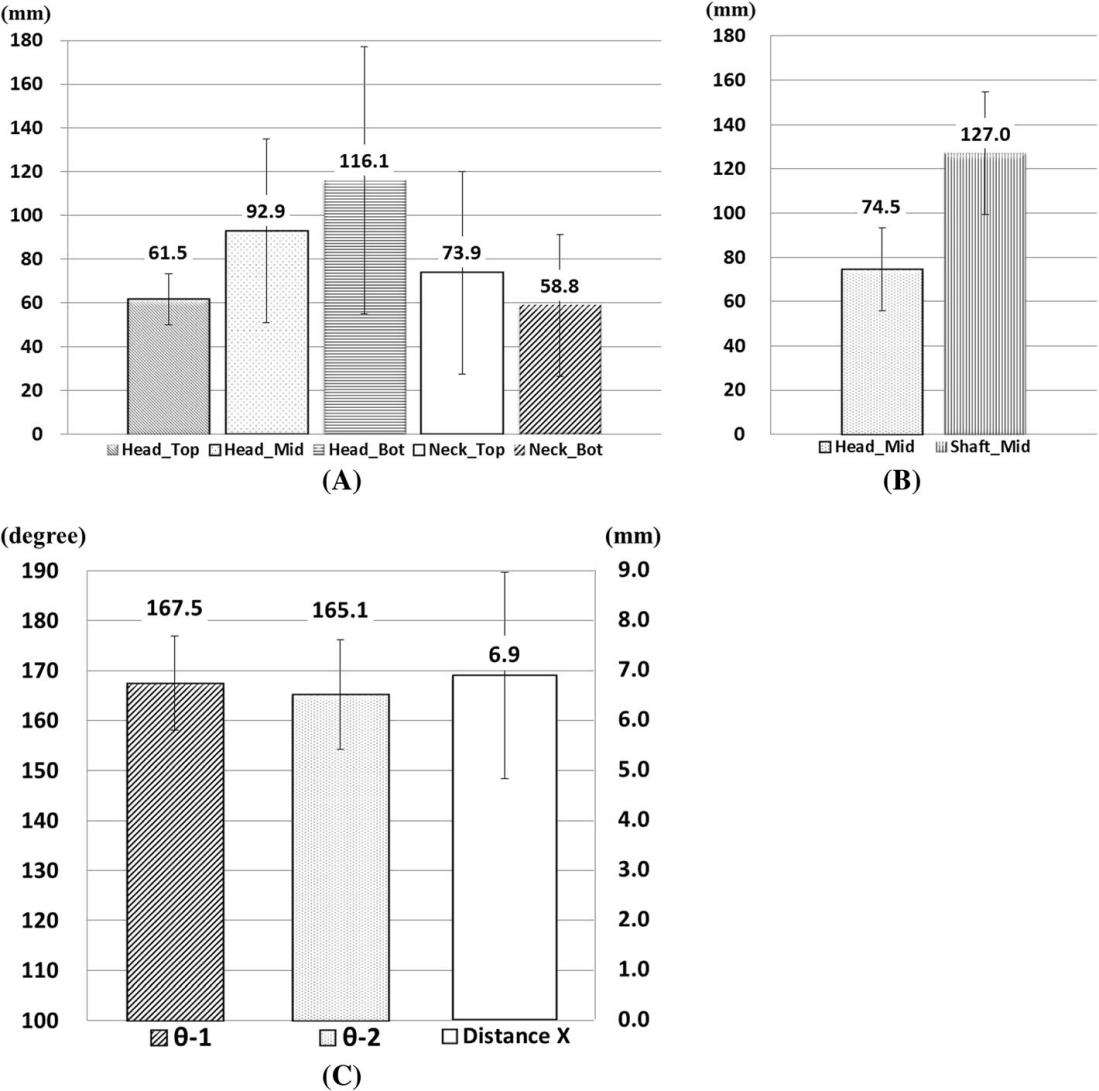
Quantitative measurement value of the radii in the axial **a** coronal plane **b** and the angle and horizontal distance of the gap **c**

The inter- and intra-observer reliabilities were satisfactory and the mean values were 0.79 (ranging from 0.68 to 0.88) and 0.76 (ranging from 0.69 to 0.84), respectively. With regard to the curvature in the axial plane, the radii of the tibial cross-sectional contour at the Head_Top, Head_Mid, and Head_Bot were 61.5 ± 11.7, 92.9 ± 42.1, and 116.1 ± 61.3 mm, respectively; there values tended to increase toward the distal direction. The radii of the tibial cross-sectional contour at the Neck_Top, and Neck_Bot were 73.9 ± 46.3 and 58.8 ± 32.4 mm, respectively; there values tended to decrease toward the distal direction (Fig. 5a). On the curvature in the coronal plane, the radii of the tibial cross-sectional contour at the Head_Mid and Shaft_Mid were 74.5 ± 18.8, 127.0 ± 27.6 mm, respectively; these values tended to increase toward the distal direction (Fig. 5b). The angles of θ–1 and θ–2 were 167.5 ± 9.4 and 165.1 ± 11.0°, respectively, and the Distance X was 6.9 ± 2.0 mm (Fig. 5c). There was no significant relationship between the θ and Distance X (*p* = n.s.).

**Fig. 6:**
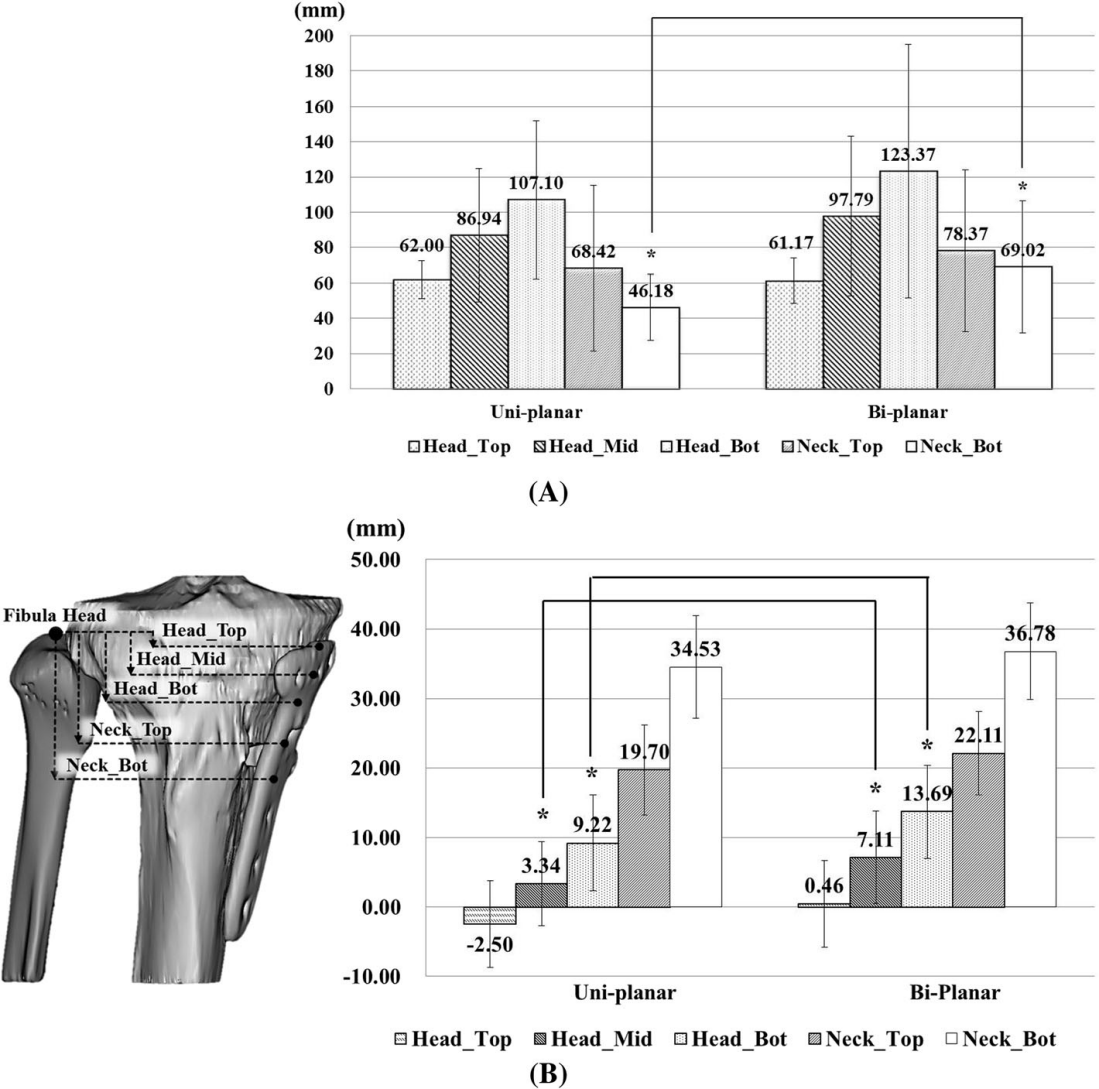
Comparison of the radii in the axial plate **a** and the distance of each cross-section from the fibular head between uni-planar and bi-planar osteotomy **b**

On comparing the values obtained for the uni-planar and bi-planar osteotomy groups, the radii of the Neck_Bot of the bi-planar group were significantly larger than that of the uni-planar group (*p* = 0.003) (Fig. 6a). Osteotomy was performed more distally in the bi-planar group than in the uni-planar group without statistical significance (Fig. 6b). Shaft_Mid and Distance X of GroupI (110.08 mm and 6.11 mm, respectively) which had lower correction angle were lower than those of GroupII (130.05 mm and 6.41 mm, respectively) and those of GroupIII (136.35 mm, 8.01 mm, respectively) in coronal plane. There were significant differences (*p* = 0.023 < 0.05 and *p* = 0.009 < 0.01, respectively). In axial plane, there was no significant difference among GroupI, GroupII, and GroupIII (Table 1).

**Table 1:** Parameters for each correction degree

		Group I (5–8 mm, *n* = 19)		Group II (9–12 mm, *n* = 29)		Group III (13–16 mm, *n* = 21)		*p*-value
		Mean	SD	Mean	SD	Mean	SD	
	Head_Top	55.8 mm	9	64.3 mm	11.8	63.4 mm	12.6	0.06
	Head_Mid	84.6 mm	37.1	103.1 mm	49.2	86.3 mm	33.9	0.23
Axial	Head_Bot	134.5 mm	89.1	115.7 mm	44	99.9 mm	32.6	0.21
	Neck_Top	70.8 mm	52.5	81.4 mm	50.7	66.4 mm	32.6	0.51
	Neck_Bot	61.8 mm	37.3	64.9 mm	34.3	47.6 mm	21.8	0.16
	Head_Mid	71 mm	15.7	78.5 mm	19.6	73 mm	21	0.58
	Shaft_Mid	110 mm	14.7	130.1 mm	16.2	136.4 mm	35.6	0.02*
Coronal	Distance X	6.1 mm	1.3	6.4 mm	1.7	8 mm	2.5	0.01*
	θ–1	166.7°	10.1	167.1°	9.6	168.7°	8.9	0.77
	θ–2	164.7°	7.5	166.1°	11.5	164.2°	13	0.81

*Abbreviation*: *SD* standard deviation

p* < 0.05

## Discussion

The principal findings of this study were (1) the radii of the tibial cross-sectional contour at the head portion tended to increase from the proximal to distal direction, (2) the radii of the tibial cross-sectional contour at the neck portion tended to decrease from the proximal to distal direction, (3) the radii of the coronal plane tended to increase from the proximal to distal direction, and (4) the angle between the proximal fragment and the distal one varied with the correction amount of the posterior opening gap. The measurements of the radii showed that the radii increased with the increase in the flatness of the surface and the radii decreased with the increase in the surface curvature. Therefore, there was an abrupt change around the posterior opening gap. In the head portion, the surface became flatter toward the distal direction. However, in the neck portion, the surface became curvier toward the distal direction on the axial plane. On the coronal plane, the surface became flatter toward the distal direction.

These changes would be expressed differently according to the correction degree and operative technique. From our results, in coronal plane, Shaft_Mid was increased in the flatness and Distance X increased as the correction amounts of the posterior opening gap increased. This could imply that it would be necessary to add an optional plate according to the correction degree. Otherwise, the plate should be bended at the both end of the opening gap in coronal plane. The radii of the uni-planar osteotomy were relatively smaller than those of the bi-planar osteotomy in the axial plane. Generally, the proximal portion has more curvature than the distal portion in the axial plane. Therefore, the level at which osteotomy was performed in the uni-planar group was relatively higher than that in the bi-planar group.

With many anthropometric studies on tibial morphologic showing several viewpoints, quantitative studies on the dimension of tibial curvature have been deficient in determining the proper fitting values for OWHTO [20–23]. It could be said that any plate failing to comply with these values did not fit well on the osteotomized bony surface of the proximal tibia, and the quantitative data of this study could provide an anatomical featured design for the OWHTO [24–26]. Proper fitting of the plate to the bony surface is the main factor in OWHTO for delivering appropriate physiological loading and making the stress path effective. Improper fitting of the plate to the bony surface may result in stress concentration of the screw and could lead to a plate fracture or a surgical failure due to loss of its fixation stability [10, 27–30].

The proximal tibia has a unique 3D anatomy as opposed to the mid or distal tibia because multiple structures change abruptly, including the tibial plateau, posterior intercondylar fossa, and posterior cortex. In addition, the center of the proximal tibia is relatively more posteriorly positioned than that of the shaft [15]. Therefore, there would be a possibility of mismatch with conventional plate positioning as described in the Fig. 1. Several anatomical changes occur additionally after OWHTO, and these changes could differ across patients, depending on various factors. The largest anatomical change is due to the primary aspect of the procedure, i.e. the opening of the wedge in the coronal plane: valgus rotation and inferior translation of the distal fragment are relative to the proximal fragment. However, other changes between the proximal and distal fragment were also noted. These secondary changes are unintended and are possibly the result of the orientation of the osteotomy cut and the hinge point relative to the long axis of the tibia [31]. Therefore, in the morphoanalysis, these changes and the morphologic characteristics around the osteotomy site should be considered simultaneously and analysis was performed with three different portions (head, neck, and shaft) in this study.

However, some limitations needed to be considered. First, only the contour of the surface was considered for the plate design. Therefore, information regarding the position of the screw and stability is lacking. Second, a detailed design of the plate has not been established. However, these data could be vital to the proper design of the OWHTO plate. Third, it is not clear how many options should be present according to the correction degree although the tendency of the change has been analyzed according to the correction degree.

## Conclusion

Current plate design should be modified to the surface geometry of the post-correction for the proper fitting. As the correction degree increases, the plate should be bended at the both end of the opening gap in coronal plane.

## Abbreviations

CT: Computed tomography
DICOM: Digital Image Communication in Medicine
HU: Hounsfield Units
OWHTO: Open wedge high tibial osteotomy
ROI: Region of interest
